# Conservative oxygen therapy in extracorporeal membrane oxygenation for post-transplant refractory hypoxemia in a pediatric liver transplant recipient with very severe hepatopulmonary syndrome: A case report

**DOI:** 10.1097/MD.0000000000045925

**Published:** 2025-11-28

**Authors:** Cheng Zeng, Jin Li, Guanghua Gao, Zhongshan Lu, Lihua Zhou, Qifa Ye, Shaojun Ye

**Affiliations:** a Zhongnan Hospital of Wuhan University, Institute of Hepatobiliary Diseases of Wuhan University; Transplant Center of Wuhan University, National Quality Control Center for Donated Organ Procurement; Key Laboratory of Medical Technology on Transplantation; Provincial Clinical Research Center for Natural Polymer Biological Liver, Wuhan, Hubei Province, China; b Department of Critical Care Medicine, Zhongnan Hospital of Wuhan University, Wuhan, China; c The 3rd Xiangya Hospital of Central South University, NHC key laboratory of translational research on transplantation medicine, Changsha, China.

**Keywords:** extracorporeal membrane oxygenation, hepatopulmonary syndrome, liver transplantation, pediatric, severe hypoxemia

## Abstract

**Rationale:**

Extracorporeal membrane oxygenation (ECMO) is an effective treatment for post-transplant refractory hypoxemia in pediatric liver transplant recipients with very severe hepatopulmonary syndrome (HPS), which has high mortality. Conservative oxygen therapy (COT) during ECMO for these patients may be helpful to avoid excessive oxygen toxicity while ensuring tissue oxygen supply. However, there is no prior report on this specific strategy. This study is the first successful report on ECMO with COT strategy in the treatment of the above-mentioned patients.

**Patient concerns:**

We report a 5-year-old patient who was admitted to the hospital due to recurrent gastrointestinal bleeding accompanied by long-term hypoxemia. The patient had a medical history of biliary atresia and had previously undergone the Kasai procedure.

**Diagnoses:**

Based on the laboratory results such as liver CT and liver function, combined with the history of Kasai operation for biliary atresia and repeated gastrointestinal bleeding, the child was diagnosed with liver failure. Upon evaluation with contrast-enhanced transthoracic echocardiography and abnormal arterial oxygenation, a diagnosis of HPS was established. Based on the preoperative arterial oxygen pressure measured at 45 mm Hg on room air, she was classified with very severe HPS.

**Interventions:**

After liver transplantation (LT), her liver function was gradually recovering. However, she required veno-arterial ECMO for severe respiratory and circulatory failure. During ECMO, COT strategy was applied.

**Outcomes:**

By the 10th day post-LT, her liver function had returned to normal completely. However, the refractory hypoxemia related to intrapulmonary arteriovenous shunting caused by HPS was not corrected immediately. Until the 38th day post-LT, with the support of the ECMO with COT strategy, HPS was completely cured.

**Lessons:**

This case demonstrates the effectiveness of combining LT and ECMO for very severe HPS, emphasizing the importance of COT combined with ECMO management.

## 1. Introduction

Hepatopulmonary syndrome (HPS) is a severe pulmonary vascular disease often associated with liver disease, leading to hypoxemia with a mortality rate of 70% in severe cases.^[1,2]^ Although liver transplantation (LT) is the most effective treatment for HPS, patients with very severe HPS still face high mortality rates post-transplant due to persistent hypoxemia caused by pulmonary vascular remodeling, infections, and edema,etc.^[1,3,4]^ Extracorporeal membrane oxygenation (ECMO) can provide temporary support in such cases.^[5]^

However, excessive oxygen administration can lead to oxygen toxicity, impair lung function, and worsen prognosis.^[6–8]^ Conservative oxygen therapy (COT) is an oxygen therapy strategy for critically ill patients in intensive care medicine. Its core principle is to prevent oxygen toxicity while ensuring adequate tissue oxygenation, through controlling blood oxygen levels within a relatively low range. The application value of this strategy in critically ill patients has been confirmed.^[6,7]^ The target indicators of COT are mainly based on arterial oxygen saturation (SaO₂) or arterial partial pressure of oxygen (PaO₂). However, the target ranges vary across studies (SaO₂ 88–98%, PaO₂ 50–100 mm Hg), and some studies adjust oxygen therapy by combining both indicators. How to balance the relationship between hypoxia and excessive oxygen exposure during COT remains controversial.^[6,8,9]^ Since it takes longer for hypoxemia to resolve in HPS, the pursuit of maintaining a normal or high oxygen target undoubtedly prolongs the duration of ECMO, increasing the risk of complications (bleeding, infection, etc).Additionally, HPS patients have long tolerated hypoxia preoperatively. Therefore, COT may be suitable for them, but there are no reported cases yet. To reduce the side effects of excessive oxygen therapy and shorten the duration of ECMO use, we adopted COT during ECMO treatment in our report. To our knowledge, for post-transplant refractory hypoxemia in a pediatric liver transplant recipient with very severe HPS, this is the first time that COT has been used during ECMO with a successful outcome.

## 2. Case presentation

This case report presents a review of the entire treatment procedure for a 5-year-old female patient who underwent LT combined with ECMO intervention at our institution due to liver failure complicated by very severe HPS. The patient had a medical history of biliary atresia and had previously undergone the Kasai procedure. Her liver disease progressed, leading to jaundice, portal hypertension, and recurrent gastrointestinal bleeding. She also experienced dyspnea, platypnea, and hypoxemia (SaO_2_ of 80%) for over a year. The physical examination upon admission showed features of chronic liver disease and cyanosis in the extremities. No manifestations of hypoxic-ischemic encephalopathy were observed. No special abnormalities were found in cardiac color ultrasound, lung CT, and head CT at admission.

**Figure 1.**
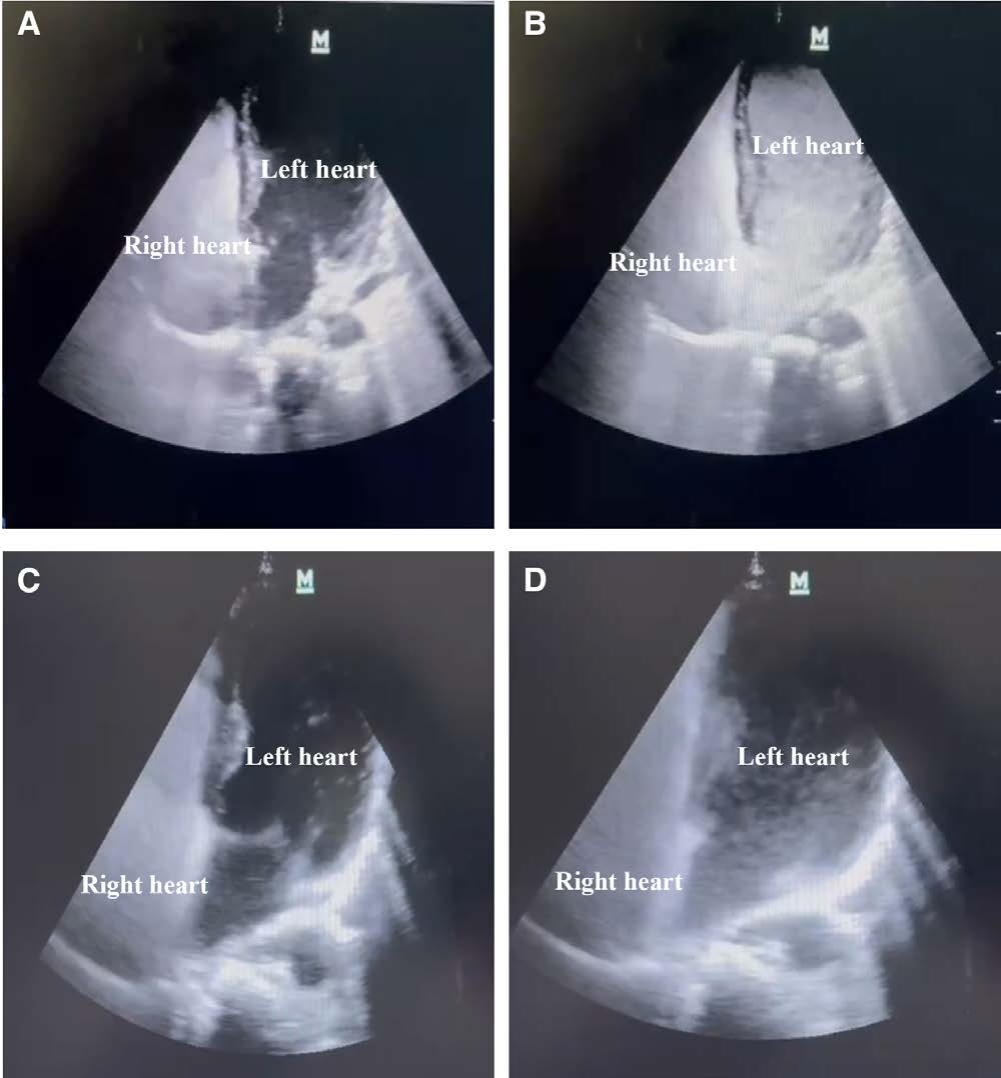
The results of preoperative and postoperative CE-TTE on day 38 (four-chamber views). Preoperative CE-TTE: (A) the beginning of intravenously injected microbubbles in the right heart; (B) after 4 cardiac cycles, a large amount of microbubbles was observed in the left heart. Postoperative CE-TTE on day 38; (C) the beginning of intravenously injected microbubbles in the right heart; (D) after 6 cardiac cycles, only a few microbubbles were observed in the left heart (details in video).

Upon evaluation with contrast-enhanced transthoracic echocardiography (CE-TTE) and abnormal arterial oxygenation1 (Fig. 1A and 1B, see Video S1), a diagnosis of HPS was established. Based on the preoperative PaO_2_ measured at 45 mm Hg on room air, she was classified with very severe HPS. Subsequently, the patient underwent whole liver transplantation from a brain-dead donor, along with methylprednisolone for immunosuppressive induction and tacrolimus for postoperative anti-rejection therapy. By the 10th day post-LT, her liver function had returned to completely normal.

However, along with the recovery of liver function, HPS had not been effectively alleviated. By the 2nd day post-LT, the patient necessitated re-intubation and mechanical ventilation due to significant hypoxemia. By the 14th day post-LT, the patient’s hypoxemia continued to worsen due to very severe HPS complicated by pulmonary infections (pneumococcal and cytomegalovirus infections) and pulmonary edema. This development precipitated severe respiratory and circulatory failure, requiring the initiation of veno-arterial (V-A) ECMO therapy (surgical vascular access for placing ECMO cannulas was achieved via the internal jugular vein and the brachiocephalic trunk artery). During ECMO support, the patient’s immune status and infection conditions were closely monitored, with corresponding adjustments made to anti-rejection and anti-infection treatment protocols. Heparin anticoagulation was used during ECMO treatment, with anticoagulation strategies adjusted based on liver function and coagulation status.

When initiating ECMO, the relevant parameters were as follows: Fraction of inspiration O_2_ (FiO_2_) 80%, rotation speed 2300 to 2700 rpm, blood flow rate 1.5 L/min, airflow rate 0.5 L/min, and SaO_2_ 85% to 90%. After 2 weeks of ECMO support, when ECMO blood flow and rotation speed were reduced to 0.9 L/min and 2100 rpm, respectively, with FiO_2_ at 40% and airflow rate at 0.5 L/min, maintaining SaO_2_ at 85% to 90%, the patient was observed for 12 hours. During this period, the patient showed good tissue perfusion and stable vital signs, allowing for successful weaning from ECMO. Following an additional one week of ventilatory assistance, utilizing FiO_2_ 40% and maintaining SaO_2_ at 85% to 90%, without any evidence of aberrant organ function or hypoxia-related tissue perfusion abnormalities, the patient was successfully weaned from mechanical ventilation and transitioned to oxygen therapy through a mask.

**Figure 2.**
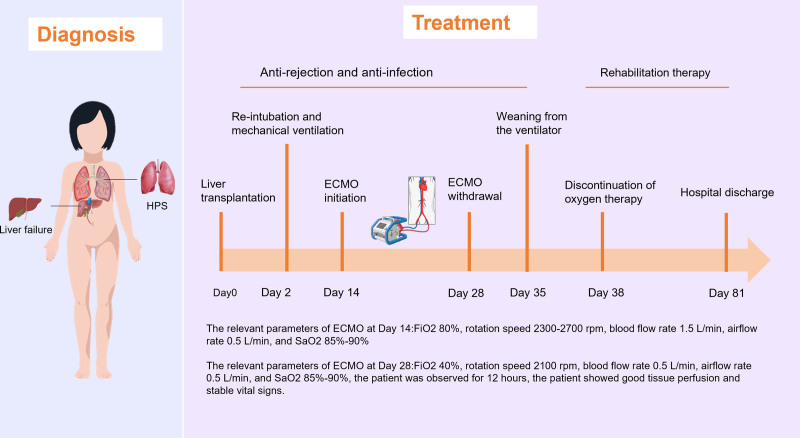
Timeline for the significant events and treatments for the patient.

By the 38th day post-transplantation, CE-TTE showed that the injected microbubbles from the right heart had nearly disappeared in the left heart (Fig. 1C and 1D, see Video S2). Concurrently, the patient’s hypoxemia had completely resolved, obviating the need for supplementary oxygen therapy. Due to severe neurological impairment caused by previous hypoxia due to severe respiratory and circulatory failure on the 14th day post-LT, the patient underwent nearly 2 months of rehabilitation therapy. By the 81st day post-transplantation, the patient was discharged with favorable outcomes, exhibiting complete restoration of neurological function, normal liver function, and no recurrence of hypoxemia (Fig. 2). The written informed consent for the treatments and the utilization of her data in our study were obtained from her parents. The study was conducted according to the 1975 Helsinki Declaration, and was approved by our Institutional Review Board. The transplantation was performed according to the Declaration of Istanbul, and no executed prisoner was used as a donor.

## 3. Discussion

HPS, caused by liver disease, involves abnormal pulmonary dilation and shunting, impairing oxygenation. LT is the best treatment, restoring liver function, reducing harmful substances (such as nitric oxide and carbon monoxide), and improving oxygenation.^[10]^ Post-transplant hypoxemia in severe HPS patients may worsen as pulmonary vascular remodeling takes time, with surgical stress and complications like infections and pulmonary edema further aggravating the condition.^[4,11,12]^

ECMO acts as a temporary life-support bridge for recovery in critically ill patients.^[5]^ High oxygen levels risk oxygen toxicity, which is harmful.^[8]^ COT aimed at minimizing harmful effects of hyperoxemia have been used successfully in these patients.^[6,7]^ HPS patients, accustomed to hypoxia, may suffer lung damage from normal oxygen levels. Additionally, the time required for pulmonary vascular remodeling varies significantly (ranging from 1 to 24 months according to current reports).^[9,10,13–19]^ Furthermore, some studies suggest that excessive oxygen intake hinders pulmonary vasoconstriction, further delaying the process of pulmonary vascular remodeling in HPS patients.^[14]^ Thus, for HPS patients without signs of tissue hypoxia, normal or high oxygen levels are unnecessary. The COT strategy can be employed instead. First of all, we use COT throughout the entire ECMO process (see the “case presentation” section for details).

Secondly, to minimize ECMO usage, we apply COT principles to ECMO mode decisions and weaning criteria. Post-V-A ECMO initiation, while hypoxemia is corrected, maintaining high oxygen levels often requires significant ECMO support, which delays weaning. In typical cases (e.g., ARDS, myocardial infarction), switching to V-V ECMO can improve oxygenation and reduce ECMO support, aiding weaning. However, this could also trigger severe hypoxemia, posing risks. There is no consensus on converting V-A to V-V ECMO for severe HPS patients.

Our experience suggests that, following COT, to avoid risks of switching from V-A to V-V ECMO, it is unnecessary to target normal or high oxygen levels. Instead, maintaining a low oxygenation state that ensures effective tissue perfusion while reducing ECMO support should be the goal for early weaning.

The timing of weaning from ECMO is also crucial. For typical patients (e.g., ARDS, myocardial infarction), criteria include normalized PaO_2_ and SaO_2_ levels, stable PaCO_2_ and pH values, cardiopulmonary function recovery, improved imaging, and stable vital signs.

To sum up, the application of COT, on one hand, helps reduce oxygen toxicity-related lung damage and promotes pulmonary vascular remodeling, while also shortening the duration of ECMO use and lowering the risk of related complications.

A review of the relevant literature revealed that there are currently 4 case reports involving the use of ECMO after liver transplantation for very severe HPS and 4 cases of other types^[9,10,14–19]^ (Table 1). There is no literature that proposes a COT strategy. Our case was weaned after 2 weeks of ECMO use, and hypoxemia was completely corrected 38 days after liver transplantation. Compared to other case reports, this represents the shortest duration, demonstrating the effectiveness of this strategy. However, this case report is constrained by the small sample size (n = 1), which limits the statistical power to draw broad conclusions. In the future, we need to increase the number of cases to verify this conclusion.

**Table 1:** Literature reviews on pediatric liver transplantation combined with ECMO treatment for HPS.

Authors	Patient age (yr)	Preoperative PaO_2_ (mm Hg)	Type of HPS	Time on ECMO (days)	ECMO configuration	Time to complete recovery from Hypoxemia after LT (days)	Prognosis
Our patient	5	45	Very severe	14	V-A	38	Alive
Kumar^[9]^	16	37	Very severe	7	V-V	More than 38 d after LT	Alive
Phillips^[10]^	1.6	67	Moderate	17	V-V	77	Alive
Huang^[14]^	13	34	Very severe	94	V-V	94	Alive
Fleming^[15]^	12	53	Severe	18	V-V	51	Alive
Jean^[16]^	1.3	N/A	N/A	9	V-A	Not applicable	Alive
Khan^[17]^	1.3	N/A	N/A	29	V-A	136	Alive
Shin^[18]^	5	37.7	Very severe	67	V-A	334	Alive
Piltcher-da-Silva^[19]^	17	34.9	Very severe	67	V-V	90	Alive

N/A = not applicable.

## 4. Conclusion

LT is the most effective treatment for HPS. However, for pediatric patients with very severe HPS, pulmonary remodeling requires time post-LT, and hypoxemia may not be immediately corrected. During the period of pulmonary vascular remodeling, the presence of concurrent pulmonary infections or pulmonary edema may directly result in respiratory failure. The timely initiation of ECMO provides crucial time for pulmonary remodeling. The application of COT strategy during ECMO is beneficial to improving prognosis.

## Author contributions

**Conceptualization:** Qifa Ye, Shaojun Ye.

**Data curation:** Cheng Zeng, Jin Li.

**Investigation:** Guanghua Gao, Zhongshan Lu.

**Project administration:** Lihua Zhou.

**Resources:** Cheng Zeng, Jin Li.

**Software:** Lihua Zhou.

**Supervision:** Qifa Ye, Shaojun Ye.

**Visualization:** Cheng Zeng.

**Writing – original draft:** Cheng Zeng, Jin Li.

**Writing – review & editing:** Qifa Ye, Shaojun Ye.
